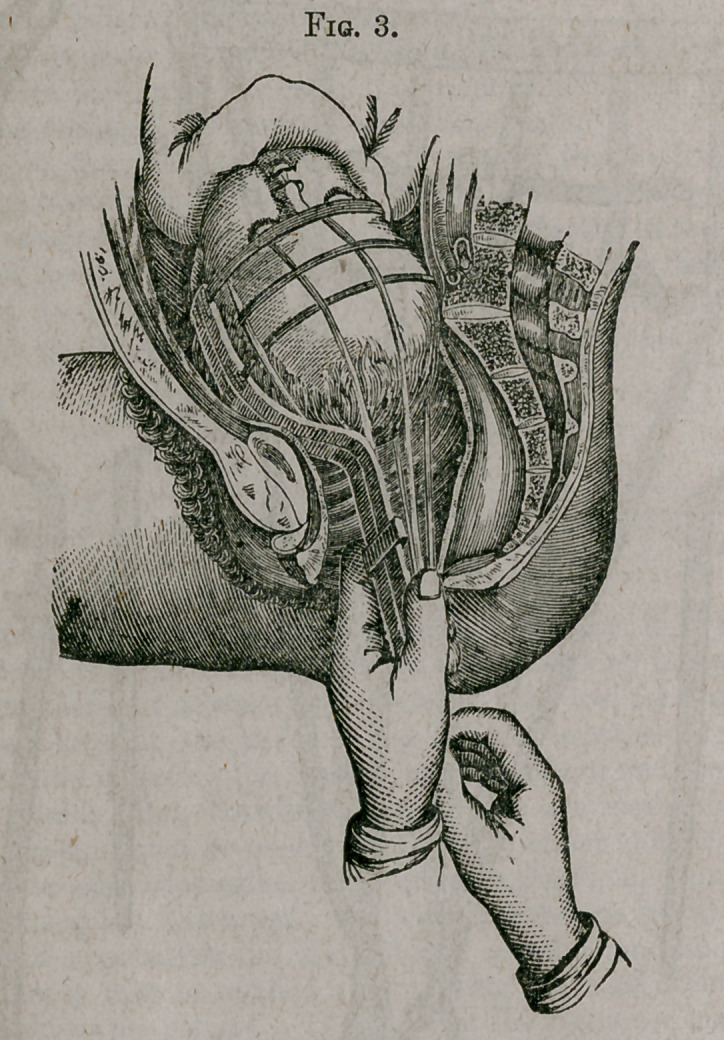# Obstetrical Extractor

**Published:** 1856-09

**Authors:** 


*Obstetrical Extractor.* By Dr. A. C. BUFFUM, Chicago, Illinois. (Two Plates.)

In presenting the Extractor to the medical profession, it is not necessary to detract from the value of the obstetrical forceps so long in use throughout the world; neither is it necessary to condemn their use, for it cannot be denied that they have, in the hands of careful and experienced accoucheurs, been instrumental in saving the lives of many mothers, and sometimes both mother and child. But, notwithstanding, there are cases often occurring where the forceps cannot be used with safety to either mother or child, and it is to fill these exigencies rather than condemn the forceps that I am desirous of introducing the Extractor. For while the forceps are so cumbersome and difficult of application, the Extractor is so small, and so simple in its application that it may be applied while making the usual examination, with little or no pain to the patient. Therefore, as the Extractor may be used in all cases, it not only does away with the use of the forceps, but furnishes the physician with an instrument that may be used in all cases where an instrument is necessary.

The principle upon which the Extractor works is plain and simple, being that of introducing two small curved and jointed steel fingers into the os tincæ upon the side of the head of the child, and circumscribing the same, placing a net cap upon the head, to which the extractive force is applied.*

The Extractor consists of two finely-polished steel fingers, each twelve

---

* It is but just to observe, that for the principle upon which the Extractor works, I am indebted to Prof. Evans, of this city; for he caused an instrument to be constructed, with two fingers, headband and down-straps, some years ago, which he introduced to the American Medical Association, and subsequently to his classes in Rush Medical College.

inches long, one-half of an inch wide, and one-eighth of an inch thick; six inches, or one-half of the length being curved and jointed, so as to fit any shaped head; to which is attached a silk braid cap, so perfect in its construction that the whole instrument, when folded, ready for application, is but one inch wide, and one-eighth of an inch thick, the cap forming a soft cushion on either side of the fingers, as seen in fig 2.

Fig. 1. Fig. 2.

Fig. 1 gives a perspective view of the instrument, A the fingers, k the joints, and j j the fenestræ in the fingers; C the principal head-band, which is nine inches long and one inch wide, extending from finger to finger, to which the down straps, i i i, are firmly stitched; D a cross band, holding the down straps in their proper position, while E is a double band, one-half inch wide, fifteen inches long, extending from finger to finger, through which the down straps pass. This band carries and holds the down straps in their proper position over the greater diameter of the head of the child. Without this stay they are liable to slip around the head whenever we attempt to make traction on either side of the head, as is necessary in rotating or moving one side at a time. F, the slide that holds the fingers in a position with each other; i i i i i i the down straps, each being eighteen inches long and one-half an inch wide, composed of sewing silk, sufficiently strong for any practical purpose. These down straps serve as the warp of the cap, and a handle to which the extractive force is applied.

Fig. 3.

To prepare the Extractor for application, double the bands C and E at h h and draw the folds through the fenestræ j j, until the fingers of the instrument are brought together, giving one-half of the net to each finger, when the slide F is to be placed upon the handles A A, and the instrument warmed in warm water and well lubricated with soap or oil.

The instrument being prepared as seen in fig. 2 for application, the patient should be placed in a comfortable position, either on her back or side, two fingers of the left hand should be introduced into the orifice of the uterus, when the instrument should be carried carefully upon the fingers into the uterus upon the side of the head, thence following the convexity of the head until the angle of the fingers of the instrument strikes the top of the head, when the slide F is to be removed and the fingers of the instrument gently separated and carried around to the opposite side of the head, until they meet, when the slide F is to be placed upon the handles, which holds them firmly together, or in other words, buckles the cross bands of the net together, as represented in fig. 3.

All is now ready for the force to be applied, but care should be taken to act in company with the labor pains, if any exist, the handles serving as a director in rotating the head, while the force is to be applied upon the down straps.

## Figures and Tables

**Fig. 1. f1:**
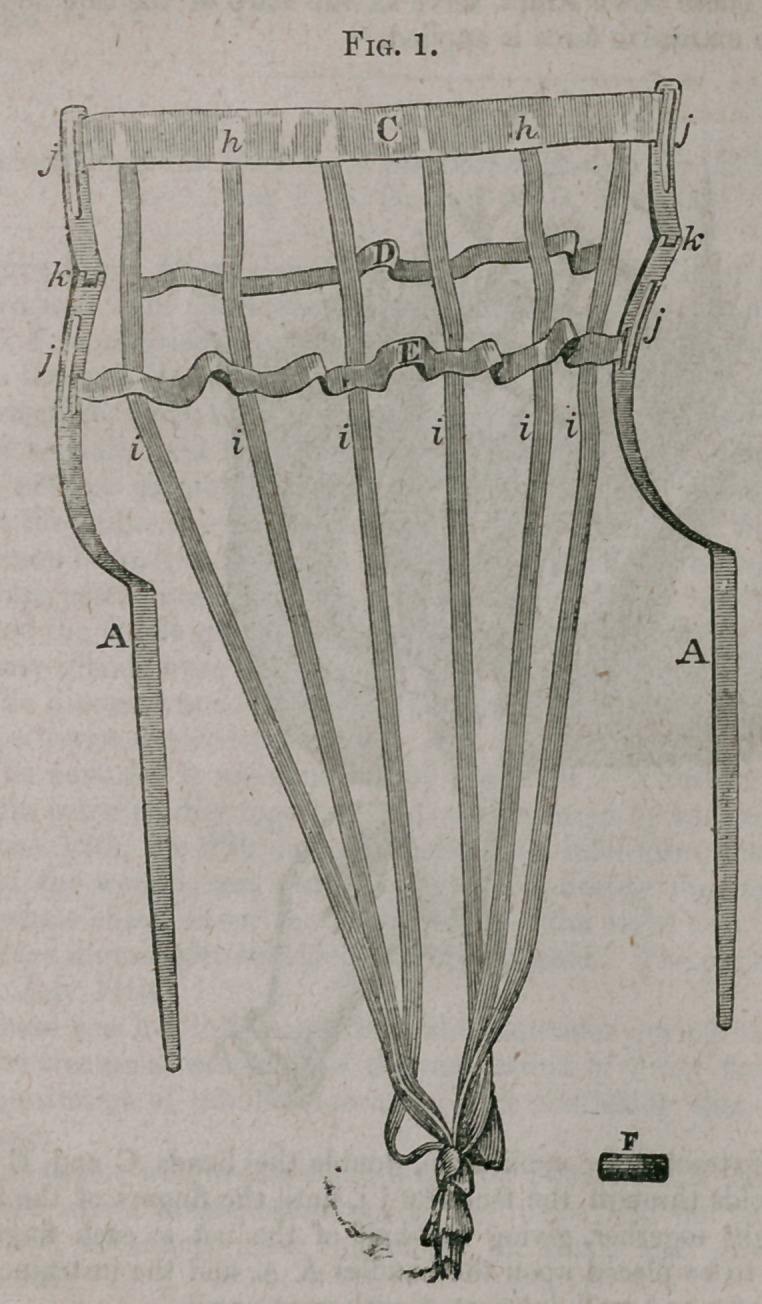


**Fig. 2. f2:**
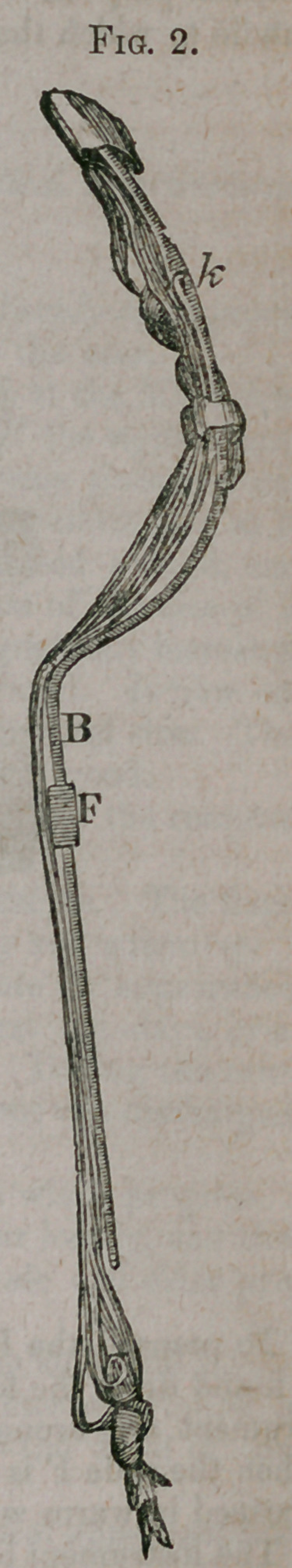


**Fig. 3. f3:**